# Oatmeal-Containing Breakfast is Associated with Better Diet Quality and Higher Intake of Key Food Groups and Nutrients Compared to Other Breakfasts in Children

**DOI:** 10.3390/nu11050964

**Published:** 2019-04-27

**Authors:** Victor L. Fulgoni, Mary Brauchla, Lisa Fleige, YiFang Chu

**Affiliations:** 1 Nutrition Impact LLC, Battle Creek, MI 49014, USA; 2 PepsiCo R&D Nutrition, Chicago, IL 60661, USA; Mary.Brauchla@pepsico.com (M.B.); Lisa.Fleige@pepsico.com (L.F.); YiFang.Chu@pepsico.com (Y.C.)

**Keywords:** whole grain, dietary fiber, soluble fiber, beta-glucan, Healthy Eating Index

## Abstract

Oatmeal is a whole grain (WG) food rich in fiber and other nutrients. The study objective was to compare diet quality and nutrient intake of children consuming oatmeal breakfasts to those of children consuming other breakfasts using the National Health and Nutrition Examination Survey (NHANES) 2011–2014. Dietary intake data from 5876 children aged 2–18 years were divided by breakfast food consumption: oatmeal breakfasts, “Doughnuts, sweet rolls, pastries”, “Pancakes, waffles, French toast”, “Eggs and omelets”, “Ready-to-eat cereal, lower sugar”, and “Ready-to-eat cereal, higher sugar” were used to assess diet quality and intake of food groups and nutrients using the USDA Healthy Eating Index-2015 (HEI-2015), Food Patterns Equivalents Database, and Food and Nutrient Database for Dietary Studies, respectively. As compared to consumers of other breakfasts or breakfast skippers, oatmeal consumers had consistently higher diet quality (4–16 points higher HEI 2015 total score, *p* < 0.05), higher WG intake (0.6–1.6 oz eq. higher, *p* < 0.05), and higher fiber and magnesium intakes compared to consumers of most other breakfasts or breakfast skippers. The results show that children consuming oatmeal breakfasts have better diet quality and increased intake of key nutrients compared to breakfast skippers and other breakfast consumers and suggest oatmeal may represent an important component of a healthy childhood diet.

## 1. Introduction

Dietary guidelines around the world unequivocally recommend increasing the consumption of whole grain. For example, the 2015–2020 Dietary Guidelines for Americans (DGA) [1] indicate that all Americans should consume half or more of their total grains as whole grains. Despite these guidelines, nearly 100% of the U.S. population across all age and gender groups does not meet the goal for whole grain intake [2]. Oats (*Avena sativa* L.) are a type of cereal grain that is considered to be a whole grain food, with commensurate levels of fiber and other key nutrients [3]. Accordingly, oats are a rich source of dietary fiber, which includes cellulose, arabinoxylans, and soluble viscous fibers, particularly β-glucan; they also have unsaturated fats and proteins, containing essential amino acids [4,5,6,7]. A 40 g serving of oats provides 152 calories, 5.3 g of protein, 27 g of carbohydrate, and 4 g of dietary fiber, and this grain is also a good source of B vitamins and minerals [8]. In addition, oats possess significant levels of antioxidants, mainly due to the presence of tocopherols, tocotrienols, phytic acid, lignins, and phenolic compounds, including avenanthramides [9,10]. Oatmeal is made from milled, steel-cut, or rolled oat grains, and some instant oatmeal products are also fortified with iron and B vitamins. Thus, oatmeal is considered to be part of a healthy diet.

The consumption of whole grains has been associated with a modestly reduced risk of cardiovascular disease, type 2-diabetes, and obesity in adults [11,12,13]. Notably, β-glucan, the soluble fiber from oatmeal, has physiological and bioactive properties that may help to improve blood lipid levels, postprandial insulin levels, glucose responses, and subjective measures of satiety [14,15,16,17,18]. The US Food and Drug Administration (FDA) has also authorized a health claim for oats/oatmeal, which states: “the consumption of 3 g or more per day of β-glucan from oats or barley may reduce the risk of coronary heart disease” [19]. Further, in a cross-sectional study, adult oatmeal consumers were reported to have better diet quality, lower body mass index (BMI), and a reduced risk of obesity as compared to non-consumers [20].

Although the benefits of oat consumption are well documented in adult populations few studies have investigated how oat intake can affect the diets of children. Oatmeal consumption by children was associated with better nutrient intake, diet quality, and reduced risk for central adiposity and obesity in a cross-sectional study [21]. However, that study did not compare oatmeal consumers with consumers of other popular breakfasts. The purpose of this study was therefore to compare diet quality and nutrient intake among children consuming an oatmeal-containing breakfast versus those of children consuming other popular breakfasts. In addition, we used dietary modeling to assess the impact of replacing other breakfast foods with oatmeal or of adding one serving of oatmeal to a percentage of children’s diets.

## 2. Methods

### 2.1. Database and Study Population

Data from the National Health and Nutrition Examination Survey (NHANES), 2011–2014, were used in all our analyses. The NHANES is an ongoing, continuous, cross-sectional survey of nutrition and health status of the US population, conducted by the National Center for Health Statistics (NCHS) in a noninstitutionalized civilian US population, using a complex, multistage, probability sampling design. Demographic and basic health information survey data are collected via in-home interviews, and comprehensive diet and health examinations are conducted in a mobile examination center. All participants or proxies provide written informed consent, and the Research Ethics Review Board at the NCHS approved the NHANES survey protocol. Detailed descriptions of the study design, interview procedures, and the physical examinations conducted are available online [22], and all data obtained from this study are publicly available at: http://www.cdc.gov/nchs/nhanes/.

Data from children aged 2 to 18 years (*n* = 7,002), participating in the NHANES from 2011 to 2012 and from 2013 to 2014 were combined to increase the sample size. Pregnant and/or lactating females and those with 24 h recall data judged to be incomplete or unreliable by the Food Surveys Research Group of the United States Department of Agriculture (USDA) (*n* = 1,126) were excluded, and, as a result, a total of 5876 children with complete information were included in our analyses. Approval from an institutional review board was not required because the study was conducted as a secondary analysis of publicly available data.

### 2.2. Breakfast Groups

Breakfast was defined as all eating occasions described as “Breakfast,” “Desayuno,” or “Almuerzo” (dr1_030z = 1, 10, 11). Consumption of quick, instant, or regular oatmeal as a cooked cereal (What We Eat in America (WWEIA) Category 4802) was classified as oatmeal breakfast, and breakfast intake of “Doughnuts, sweet rolls, pastries” (WWEIA Category 5506), “Pancakes, waffles, French toast” (WWEIA Category 4404), “Eggs and omelets” (WWEIA Category 2502), “Ready-to-eat cereal, lower sugar” (sugar ≤ 21.2 g/100 g; WWEIA Category 4604), or “Ready-to-eat cereal, higher sugar” (sugar > 21.2 g/100 g; WWEIA Category 4602) was defined as other breakfast [23]. For dietary modeling analyses, two different types of modifications were used: (a) other breakfast foods were replaced with oatmeal, and (b) a reference amount customarily consumed (RACC) or 40 g of dry oatmeal as prepared was added to the diet. Modifications were made to 10, 20, and 30% of sample population diets, and nutrient content of “Oatmeal, cooked, regular, quick or instant, fat not added in cooking” (food-codes 56203000, 56203010, and 56203020) was used for dietary modeling.

### 2.3. Intake Estimates

Dietary intake data were obtained from in-person 24 h dietary recall interviews that were administered using an automated, multiple-pass method [22]. However, only data from Day 1 dietary recalls were used in this study to ensure methodological consistency. Parents or guardians provided 24 h dietary recall for children 2–5 years of age and assisted recalls for children 6–11 years of age, whereas older children provided their own recalls. Primary outcome measures were based on diet quality and intake of specific food groups or nutrients of interest. Diet quality scores were determined using the USDA Healthy Eating Index-2015 (HEI-2015) [24]. Food groups of interest included whole grains, refined grains, and dairy, and these were calculated using the USDA Food Patterns Equivalents Database (FPED) [25]. Energy and nutrient intake were determined using the USDA Nutrient Database for Standard Reference Releases [26] in conjunction with the respective Food & Nutrient Database for Dietary Studies for each NHANES cycle [27,28], and nutrients of interest were selected on the basis of current dietary recommendations [1]. The DGA has identified iron, magnesium, folic acid, and vitamins A, C, and E as “shortfall nutrients”, as these are currently under-consumed, and has classified fiber, calcium, potassium, and vitamin D as “nutrients of public health concern” due to the fact that current intakes of these are so low as to pose specific health concerns [1].

### 2.4. Statistics

We used SAS 9.4 (SAS Institute, Cary, NC, USA) and SUDAAN 11 (RTI, Research Triangle Park, NC, USA) software for all statistical analyses. The data were adjusted for the complex sampling design of NHANES, using appropriate survey weights, strata, and primary sampling units. Day 1 dietary weights were used in all intake analyses. Least-square means (LSM) and standard errors (SE) were generated via regression analyses for diet quality, as well as food group, energy, and nutrient intake. Intake data were adjusted for age, gender, race/ethnicity, poverty income ratio (<135, 135–185, and >185% of poverty level), physical activity level (defined as sedentary, moderate, or vigorous on the basis of responses to physical activity questionnaire), and kcal (except for energy intake and HEI).

## 3. Results

### 3.1. Demographics

On the day of dietary recall, only 173 (2.94%) children 2–18 years of age reported consuming oatmeal at breakfast. We found no significant gender differences among the consumers of different breakfasts. However, we did detect significant differences in mean age, ethnicity, poverty income ratio, and physical activity between children who consumed different breakfasts, thus indicating the need to use these variables as covariates in subsequent analyses. Specifically, we found that compared to other breakfast consumers, oatmeal consumers were 1–3 years younger. This group also contained the lowest proportion of Hispanics, as well as the highest proportion of both Non-Hispanic (NH) blacks, and those involved in vigorous physical activity (Table 1).

### 3.2. Comparison with Breakfast skippers

We further found that child consumers of oatmeal breakfasts had a 30% higher diet quality (HEI 2015 total score), as compared to those who skipped breakfast (Table 2). Intake of key food groups, such as dairy (+40.7%), whole grain (+265%), and fruit (+53.3%) was also higher in the oatmeal-consuming group, whereas intake of refined grain was lower (−14.6%), as compared to breakfast skippers. Additionally, children who consumed oatmeal breakfasts had a significantly higher intake of energy (+18.4%), protein (+9.9%), fiber (+44%), calcium (+33.5%), iron (+21.4%), magnesium (+37.9%), potassium (+21.8%), folate (+13.9%), vitamin A (+52.3%), and vitamin D (+52.2%) than children who skipped breakfast (Table 2).

### 3.3. Comparison with Other Breakfasts

We next compared oatmeal breakfast consumers with children who consumed other breakfasts and found that oatmeal consumers had significantly higher diet quality (HEI 2015 total score) and whole grain intake compared to consumers of all other breakfasts investigated, including “Doughnuts, sweet rolls, pastries”, “Pancakes, waffles, French toast”, “Eggs and omelets”, and “Ready-to-eat cereals, lower and higher sugar” (Table 3). Additionally, oatmeal consumers had higher intake of fiber, calcium, iron, magnesium, potassium, and vitamins A and E than consumers of “Doughnuts, sweet rolls, pastries”; higher intake of magnesium and potassium than consumers of “Pancakes, waffles, French toast”; higher intakes of fiber, calcium, iron, and magnesium than consumers of “Eggs and omelets”; and higher intake of fiber than consumers of “Ready-to eat-cereal, higher sugar”. However, intake of both iron and folate was lower among oatmeal consumers compared to consumers of “Ready-to-eat cereal, lower and higher sugar” (Table 3).

### 3.4. Dietary Modeling

Lastly, we investigated the effects of replacing other breakfast foods with oatmeal, as well as of adding one RACC serving of oatmeal, via dietary modeling. From these analyses, we observed few effects on dietary intake associated with the replacement of other breakfast foods with oatmeal. Specifically, only the intakes of whole grain and magnesium increased (+33% and +4%, respectively) due to the replacement of other breakfast foods with oatmeal in 30% of the population, whereas folate intake decreased (−6%) (Table 4 and Table 5). However, the addition of one oatmeal serving in 30% of the population modestly increased overall diet quality (HEI 2015 total score increased from 48.6 to 50.1). Intake of whole grain, fiber, and magnesium was also increased (+51%, +8%, and +7%, respectively) by the addition of one serving of oatmeal to the diets of 30% of the population (Table 4 and Table 5). 

## 4. Discussion

In this cross-sectional analysis of data from the NHANES 2011–2014, we found that children consuming oatmeal at breakfast had better overall diet quality, as well as a higher intake of whole grain, fiber, and a number of micronutrients, than children consuming other popular breakfasts or those who skipped breakfast. Notably, oatmeal consumption was also associated with better nutrient intake and diet quality in our earlier analysis of the NHANES 2001–2010 data, when comparing the diets of child and adult oatmeal consumers with those of non-consumers [20,21]. However, this current study represents the first cross-sectional analysis of NHANES data comparing the diets of children consuming oatmeal breakfasts with those of children consuming other common breakfasts.

From our analyses of the NHANES data, we found that children consuming oatmeal for breakfast had significantly higher overall diet quality than children consuming either no breakfast or other common breakfasts. These results are consistent with our earlier analysis of the NHANES 2001–2010 data, which found that consumers of oatmeal have a significantly higher diet quality than non-consumers [20,21]. HEI is a validated measure of diet quality commonly used to evaluate diets [29,30,31], to assess the efficacy of dietary interventions, and to validate other nutrition research tools and indexes [32]. It has also been used in recent research to understand the relationships between nutrients/foods/dietary patterns and health-related outcomes [33,34,35,36]. With this metric, a higher score is indicative of compliance/adherence to dietary recommendations using 13 components (nine for adequacy and four for moderation), each of which relates to key recommendations of the DGA [1]. Here, we found that the HEI 2015 total scores of oatmeal consumers were significantly higher than those of breakfast skippers or consumers of other breakfasts, indicating higher compliance to nutritional guidelines, but oatmeal consumers still have room for improvement in their diets. However, there were differences in the mean age among consumers of different breakfasts and breakfast skippers, and, while we did adjust for age, it is possible that this age difference might still have influenced the dietary behaviors. 

Oatmeal is a whole grain breakfast, and, in accordance with this, we found that child consumers of oatmeal had a significantly higher intake of whole grain than consumers of other breakfasts or breakfast skippers. In spite of public health efforts to increase whole grain intake, research studies have shown that consumption among children and adolescents remains low [37,38,39,40], and nearly 100% of children consume less than the recommended amount (i.e., 1.5 oz. eq. for younger children and 3–4 oz. eq. for older children and adolescents) [2]. This deficient intake of whole grains leads to under-consumption of several shortfall nutrients and nutrients of public health concern, including fiber [2]. In the present study, consumers of oatmeal breakfasts were found to have a 72% higher whole grain intake than breakfast skippers, and a 30–80% higher intake than consumers of other breakfasts. These intake estimates are also similar to our earlier findings from previous NHANES data [21].

Oatmeal is one of the richest sources of the fiber β-glucan, which has been associated with improvement in blood cholesterol and postprandial glycemic and insulinemic responses, as well as with reduced risk of coronary heart disease (CHD) and increased satiety in adults [14,15,16,17,18,19]. In this study, we found that oatmeal consumers had a significantly higher intake of dietary fiber than breakfast skippers or consumers of “Doughnuts, sweet rolls, pastries”, “Eggs and omelets”, and “Ready-to-eat cereal, higher sugar”. The global health effects of dietary fiber have not been extensively investigated in children. However, it has been well established that increased fiber intake is associated with better diet quality and lower risk of overweight/obesity in both adults and children [1,20,21,41], and, therefore, the consumption of oatmeal and other fiber-rich foods should be promoted.

Here, we further found that child oatmeal consumers had significantly higher intake of fiber, calcium, iron, potassium, and magnesium than breakfast skippers or consumers of a number of the other breakfasts studied. All of these nutrients are currently under-consumed and have been identified as “shortfall nutrients” by the DGA [1]. In addition, the DGA has classified calcium, potassium, and fiber as “nutrients of public health concern” due to the fact that their current intakes are quite low and may pose a public health concern [1]. Thus, foods containing these nutrients should be promoted, particularly for children. 

Somewhat surprisingly, however, based on our dietary modeling experiments, the effects of replacing other breakfasts with oatmeal or of adding one RACC of oatmeal in up to 30% of the population were largely insignificant, with a few exceptions. The reasons for this are unclear, although, as one of the four meals of the day (breakfast, lunch, dinner, and snacks), breakfast generally represents about one-fourth of daily energy and nutrient intake; therefore, a change in breakfast for 30% population may not be large enough to have a statistically significant effect. 

The strengths of this study include the use of a large, nationally representative population sample. However, our conclusions are limited to associations, and thus the elucidation of cause-and-effect relationships must be assessed in future studies. In addition, 24 h dietary recalls, which are utilized in the NHANES, rely on the memory of participants for self-reported dietary intake and are therefore subject to under- and over-reporting and may not represent long-term intake. Given that oatmeal may have been among other foods consumed concomitantly, the differences reported here might not be solely attributable to oatmeal consumption at breakfast. The present study did not make any differentiation between regular oatmeal and oatmeal with added sugars.

## 5. Conclusions

In conclusion, the results from this study suggest that children consuming oatmeal at breakfast have a better diet quality and an increased intake of a number of key nutrients, as compared to breakfast skippers and consumers of other popular breakfasts. These data therefore suggest that oatmeal may represent an important component of a healthy childhood diet. Additional studies that further evaluate the health benefits of consuming high-fiber foods, such as oatmeal, are needed to better understand the ways in which whole grains promote health in children.

## Figures and Tables

**Table 1:** Demographic profiles of child consumers of different breakfasts and breakfast skippers, ages 2–18 years, National Health and Nutrition Examination Survey (NHANES) 2011–2014.

	Breakfast Skippers	Oatmeal	Doughnuts, Sweet Rolls, Pastries	Pancakes, Waffles, French Toast	Eggs and Omelets	Ready-to-Eat Cereal, Lower Sugar	Ready-to-Eat Cereal, Higher Sugar	*p*-Value *
Age, years	13.1 ± 0.2	7.14 ± 0.72	10.4 ± 0.4	8.63 ± 0.23	9.72 ± 0.37	8.77 ± 0.48	9.04 ± 0.17	<0.0001
Gender								
Male, %	47.8 ± 2.5	56.5 ± 5.1	54.7 ± 3.2	54.4 ± 3.4	55.0 ± 2.6	51.2 ± 4.0	52.1 ± 2.1	0.1720
Ethnicity								
Hispanic, %	22.5 ± 3.3	14.1 ± 4.1	19.8 ± 3.4	19.0 ± 3.2	31.2 ± 4.2	26.5 ± 4.7	21.9 ± 2.8	0.0021
NH-white, %	49.1 ± 5.2	57.6 ± 6.3	66.4 ± 4.8	61.5 ± 4.5	44.6 ± 5.7	58.2 ± 5.6	53.7 ± 4.2	0.0001
NH-black, %	21.1 ± 3.7	20.3 ± 5.9	7.83 ± 2.01	11.8 ± 2.0	14.6 ± 2.6	6.36 ± 1.65	14.6 ± 2.1	0.0002
Asian, %	4.6 ± 0.8	2.99 ± 1.36	1.33 ± 0.46	2.66 ± 0.80	3.04 ± 0.78	3.71 ± 1.03	4.52 ± 1.14	0.0126
Other, %	2.7 ± 0.5	5.02 ± 2.71	4.66 ± 1.95	4.97 ± 1.34	6.52 ± 2.29	5.30 ± 1.51	5.35 ± 1.02	0.0602
Poverty Income Ratio								
<1.35, %	43.3 ± 4.0	37.1 ± 7.1	32.5 ± 4.1	27.7 ± 3.7	41.0 ± 4.0	38.2 ± 4.9	41.9 ± 3.7	0.0019
1.35–1.85, %	13.5 ± 1.6	12.6 ± 4.7	15.7 ± 4.0	8.57 ± 1.59	10.5 ± 2.8	10.2 ± 2.2	11.2 ± 1.7	0.1651
>1.85 (%)	43.2 ± 3.9	50.3 ± 7.6	51.8 ± 4.9	63.7 ± 3.9	48.5 ± 4.8	51.6 ± 5.4	46.9 ± 3.8	0.0004
Physical Activity								
Sedentary, %	11.0 ± 2.0	4.70 ± 1.95	10.1 ± 2.3	12.6 ± 2.7	10.8 ± 1.8	7.73 ± 1.68	9.25 ± 1.76	0.0708
Moderate, %	29.1 ±2.2	11.1 ± 2.8	19.7 ± 2.1	20.0 ± 3.0	20.7 ± 3.6	13.2 ± 3.0	19.8 ± 1.4	0.0010
Vigorous, %	60.0 ± 3.2	84.2 ± 3.6	70.1 ± 3.2	67.5 ±3.7	68.6 ± 3.4	79.1 ± 2.5	71.0 ± 2.1	0.0001

Data are presented as sample-weighted means ± standard error (SE); NH, non-Hispanic; * Test of equal means across seven breakfasts groups.

**Table 2:** Comparison of diet quality and intake of select food groups and nutrients for children 2–18 years of age (gender-combined), who consumed oatmeal breakfasts and those who skipped breakfast, NHANES 2011–2014.

	Oatmeal (*n* = 173)	Breakfast Skippers (*n* = 894)	Difference	*p*-Value
HEI 2015	58.6 ± 1.2	45.1 ± 0.7	13.5 ± 1.6	<0.0001
Dairy (cup eq.)	2.56 ± 0.26	1.82 ± 0.10	0.74 ± 0.27	0.0092
Whole Grain (oz. eq.)	2.01 ± 0.19	0.55 ± 0.07	1.46 ± 0.21	<0.0001
Refined Grain (oz. eq.)	4.86 ± 0.33	5.69 ± 0.16	−0.83 ± 0.35	0.0250
Fruit (cup eq.)	1.41 ± 0.19	0.92 ± 0.11	0.49 ± 0.23	0.0443
Energy (kcal)	2026 ± 67	1711 ± 48	315 ± 84	0.0007
Protein (g)	71.1 ± 2.9	64.7 ± 1.3	6.45 ± 3.01	0.0399
Fiber (g)	18.0 ± 0.8	12.5 ± 0.4	5.53 ± 0.74	<0.0001
Calcium (mg)	1187 ± 68	889 ± 27	298 ± 79	0.0007
Iron (mg)	13.6 ± 0.6	11.2 ± 0.2	2.44 ± 0.65	0.0007
Magnesium (mg)	295 ± 7	214 ± 4	81.3 ± 8.4	<0.0001
Potassium (mg)	2451 ± 99	2013 ± 52	437 ± 113	0.0005
Folate (µg)	320 ± 17	281 ± 10	39.2 ± 18.8	0.0449
Vitamin A, RAE (µg)	699 ± 51	459 ± 22	240 ± 55	0.0001
Vitamin C (mg)	80.5 ± 6.5	71.1 ± 12.0	9.34 ± 14.08	0.5116
Vitamin D (µg)	6.24 ± 0.75	4.10 ± 0.20	2.13 ± 0.82	0.0136
Vitamin E (mg)	7.45 ± 0.38	6.67 ± 0.23	0.78 ± 0.44	0.0836

Data were adjusted for the following covariates: age, gender, ethnicity, poverty income ratio, physical activity, and kcal (except for energy), and are presented as the mean ± SE; HEI, USDA Healthy Eating Index-2015; RAE, Retinol Activity Equivalents.

**Table 3:** Differences in diet quality and intake of select food groups and nutrients for children 2–18 years of age (gender-combined), who consumed oatmeal breakfasts compared to those who consumed other common breakfasts, NHANES 2011–2014.

Difference between Consumers of Oatmeal Breakfast and other Common Breakfasts ^a^					
	Doughnuts, Sweet Rolls, Pastries (*n* = 357)	Pancakes, Waffles, French Toast (*n* = 636)	Eggs and Omelets (*n* = 720)	Low-Sugar Ready-to-Eat Cereal (*n* = 424)	High-Sugar Ready-to-Eat Cereal (*n* = 1336)
HEI 2015	16.4 ± 1.6 **	11.3 ± 1.3 **	10.8 ± 1.5 **	3.65 ± 1.60 *	8.87 ± 1.30 **
Dairy (cup eq.)	0.60 ±0.31	0.47 ± 0.26	0.65 ± 0.26 *	−0.11 ± 0.26	0.07 ± 0.24
Whole Grain (oz. eq.)	1.61 ± 0.20 **	1.30 ± 0.22 **	1.27 ± 0.23 **	0.60 ± 0.22 *	1.08 ± 0.19 **
Refined Grain (oz. eq.)	−2.01 ± 0.39 **	−1.71 ± 0.36 **	−0.56 ± 0.39	−0.66 ± 0.40	−0.91 ± 0.35 *
Fruit (cup eq.)	0.60 ± 0.25 *	0.30 ± 0.17	0.26 ± 0.20	0.23 ± 0.18	0.33 ± 0.18
Energy (kcal)	-241 ± 90 *	−41.2 ± 75.6	4.26 ± 84.99	132 ± 90	107 ± 71
Protein (g)	11.3 ± 4.3 *	6.67 ± 3.11 *	−4.83 ± 3.09	−6.45 ±3.65	3.56 ± 3.07
Fiber (g)	6.13 ± 0.76 **	4.21 ± 0.88 **	5.19 ± 0.87 **	0.71 ±1.12	3.42 ± 0.74 **
Calcium (mg)	228 ± 95 *	131 ± 74	201 ± 72 **	−40.8 ± 78.0	38.8 ± 66.7
Iron (mg)	1.67 ±0.81 *	−0.26 ± 0.57	1.34 ± 0.64 *	−9.82 ± 1.27 **	−3.41 ± 0.75 **
Magnesium (mg)	96.1 ± 10.9 **	68.5 ± 7.4 **	59.1 ± 8.3 **	10.3 ± 14.3	52.1 ± 7.8 **
Potassium (mg)	572 ± 142 **	341 ± 101 **	211 ± 107	−108 ± 123	197 ± 107
Folate (µg)	−13.5 ± 18.6	−11.9 ± 16.2	6.18 ± 18.62	−257 ± 31 **	−157 ± 20 **
Vitamin A, RAE (µg)	213 ± 73 **	−46.1 ± 58.2	88.2 ± 52.7	−95.1 ± 61.7	−31.7 ± 46.2
Vitamin C (mg)	20.4 ± 10.5	7.13 ± 7.2	−2.89 ± 9.08	−8.86 ± 9.02	0.20 ± 7.62
Vitamin D (µg)	1.54 ± 0.98	0.97 ± 0.83	0.06 ± 0.86	−1.61 ± 0.82	−1.20 ± 0.74
Vitamin E (mg)	1.60 ± 0.56 *	0.64 ± 0.44	0.34 ± 0.39	−1.72 ± 1.80	0.65 ± 0.42

Data were adjusted for the following covariates: age, gender, ethnicity, poverty income ratio, physical activity, and kcal (except for energy) and are presented as the mean ± SE; * *p* < 0.05 and ** *p* < 0.01 compared to the oatmeal group. ^a^ Children consuming oatmeal breakfast in addition to other common breakfasts were excluded from both groups.

**Table 4:** Effects of replacing other breakfasts with oatmeal on diet quality and on the intake of select food groups and nutrients among children 2–18 years of age (gender-combined), NHANES 2011–2014.

	No Modification	Modification in 10% of the Population	Modification in 20% of the Population	Modification in 30% of the Population
HEI 2015	48.6 (47.9, 49.3)	48.9 (48.2, 49.6)	49.3 (48.6, 50.0)	49.6 (48.9, 50.3)
Dairy (cup eq.)	2.18 (2.10, 2.26)	2.18 (2.10, 2.26)	2.18 (2.10, 2.25)	2.17 (2.10, 2.25)
Whole Grain (oz. eq.)	0.78 (0.72, 0.84)	0.87 (0.81, 0.92)	0.95 (0.89, 1.01) *	1.04 (0.98, 1.10) *
Refined Grain (oz. eq.)	5.89 (5.73, 6.05)	5.85 (5.68, 6.01)	5.80 (5.64, 5.97)	5.76 (5.59, 5.92)
Fruit (cup eq.)	1.10 (1.03, 1.17)	1.10 (1.03, 1.17)	1.10 (1.03, 1.17)	1.10 (1.03, 1.17)
Energy (kcal)	1902 (1866, 1937)	1902 (1866, 1937)	1902 (1866, 1937)	1902 (1866, 1937)
Protein (g)	68.7 (66.9, 70.4)	68.8 (67.0, 70.5)	68.8 (67.1, 70.6)	68.9 (67.2, 70.7)
Fiber (g)	14.0 (13.7, 14.3)	14.2 (13.9, 14.5)	14.4 (14.1, 14.7)	14.6 (14.3, 14.9)
Calcium (mg)	1044 (1016, 1072)	1041 (1013, 1069)	1038 (1010, 1066)	1035 (1007, 1063)
Iron (mg)	14.0 (13.6, 14.3)	13.8 (13.4, 14.1)	13.6 (13.2, 13.9)	13.4 (13.0, 13.7)
Magnesium (mg)	236 (231, 240)	239 (234, 243)	242 (237, 246)	245 (240, 249) *
Potassium (mg)	2195 (2149, 2241)	2199 (2152, 2245)	2202 (2156, 2249)	2206 (2160, 2252)
Folate (µg)	367 (356, 377)	359 (349, 370)	352 (342, 362)	345 (335, 355) *
Vitamin A, RAE (µg)	596 (577, 615)	585 (566, 604)	574 (556, 593)	564 (545, 582)
Vitamin C (mg)	76.8 (71.9, 81.7)	76.5 (71.6, 81.4)	76.2 (71.3, 81.1)	75.9 (71.0, 80.8)
Vitamin D (µg)	5.67 (5.46, 5.88)	5.61 (5.40, 5.82)	5.55 (5.34, 5.76)	5.49 (5.28, 5.70)
Vitamin E (mg)	7.05 (6.71, 7.39)	7.01 (6.68, 7.34)	6.97 (6.66, 7.28)	6.93 (6.63, 7.23)

Data are presented as the mean (95% confidence interval [CI]); * Indicates meaningful differences (non-overlapping 95% CI) from no modification.

**Table 5:** Effect of adding one reference amount customarily consumed (RACC) of oatmeal on diet quality and on the intake of select food groups and nutrients among children 2–18 years of age (gender-combined), NHANES 2011–2014.

	No Modification	Modification in 10% of the Population	Modification in 20% of the Population	Modification in 30% of the Population
HEI 2015	48.6 (47.9, 49.3)	49.1 (48.4, 49.8)	49.6 (48.9, 50.3)	50.1 (49.4, 50.8) *
Dairy (cup eq.)	2.18 (2.10, 2.26)	2.18 (2.10, 2.26)	2.18 (2.10, 2.26)	2.18 (2.10, 2.26)
Whole Grain (oz. eq.)	0.78 (0.72, 0.84)	0.91 (0.85, 0.97) *	1.04 (0.99, 1.10) *	1.18 (1.12, 1.24) *
Refined Grain (oz. eq.)	5.89 (5.73, 6.05)	5.89 (5.73, 6.05)	5.89 (5.73., 6.05)	5.89 (5.73, 6.05)
Fruit (cup eq.)	1.10 (1.03, 1.17)	1.10 (1.03, 1.17)	1.10 (1.03, 1.17)	1.10 (1.03, 1.17)
Energy (kcal)	1902 (1866, 1937)	1916 (1881, 1951)	1930 (1895, 1966)	1944 (1909, 1980)
Protein (g)	68.7 (66.9, 70.4)	69.2 (67.4, 70.9)	69.7 (67.9, 71.4)	70.2 (68.4, 71.9)
Fiber (g)	14.0 (13.7, 14.3)	14.4 (14.1, 14.7)	14.8 (14.4, 15.1) *	15.1 (14.8, 15.5) *
Calcium (mg)	1044 (1016, 1072)	1046 (1018, 1075)	1049 (1021, 1077)	1051 (1023, 1080)
Iron (mg)	14.0 (13.6, 14.3)	14.1 (13.7, 14.5)	14.3 (13.9, 14.6)	14.4 (14.4, 14.8)
Magnesium (mg)	236 (231, 240)	241 (236, 246)	246 (242, 251) *	252 (247, 256) *
Potassium (mg)	2195 (2149, 2241)	2208 (2162, 2255)	2221 (2175, 2268)	2234 (2188, 2281)
Folate (µg)	367 (356, 377)	367 (357, 378)	368 (358, 379)	369 (359, 380)
Vitamin A, RAE (µg)	596 (577, 615)	596 (577, 615)	596 (577, 615)	596 (577, 615)
Vitamin C (mg)	76.8 (71.9, 81.7)	76.8 (71.9, 81.7)	76.8 (71.9, 81.7)	76.8 (71.9, 81.7)
Vitamin D (µg)	5.67 (5.46, 5.88)	5.67 (5.46, 5.88)	5.67 (5.46, 5.88)	5.67 (5.46, 5.88)
Vitamin E (mg)	7.05 (6.71, 7.39)	7.07 (6.73, 7.41)	7.08 (6.75, 7.42)	7.10 (6.76, 7.44)

Data are presented as the mean (95% confidence interval [CI]). * Indicates meaningful differences (non-overlapping 95% CI) from no modification.

## References

[1] Dietary Guidelines for Americans. 2015–2020, 8th Edition. http://health.gov/dietaryguidelines/2015/guidelines/.

[2] Scientific Report of the 2015 Dietary Guidelines Advisory Committee: Advisory Report to the Secretary of Health and Human Services and the Secretary of Agriculture. https://health.gov/dietaryguidelines/2015-scientific-report/pdfs/scientific-report-of-the-2015-dietary-guidelines-advisory-committee.pdf.

[3] The Whole Grain Council. http://wholegrainscouncil.org.

[4] Ryan D., Kendall M., Robards K. (2007). Bioactivity of oats as it relates to cardiovascular disease. Nutr. Res. Rev..

[5] Drzikova B., Dongowski G., Gebhardt E., Habel A. (2005). The composition of dietary fibre-rich extrudates from oat affects bile acid binding and fermentation in vitro. Food Chem..

[6] Welch R., Brown J., Leggett J. (2000). Interspecific and intraspecific variation in grain and groat characteristics of wild oat (Avena) species: Very high groat (1-3)(1-4)β-D-glucan in an Avena atlantica genotype. J. Cereal. Sci..

[7] Clemens R., van Klinken B.J. (2014). The future of oats in the food and health continuum. Br. J. Nutr..

[8] United States Department of Agriculture Agricultural Research Service National Nutrient Database for Standard Reference. https://www.ars.usda.gov/northeast-area/beltsville-md-bhnrc/beltsville-human-nutrition-research-center/nutrient-data-laboratory/docs/usda-national-nutrient-database-for-standard-reference/.

[9] Rasane P., Jha A., Sabikhi L., Kumar A., Unnikrishnan V.S. (2015). Nutritional advantages of oats and opportunities for its processing as value added foods—A review. J. Food Sci. Technol..

[10] Perrelli A., Goitre L., Salzano A.M., Moglia A., Scaloni A., Retta S.F. (2018). Biological activities, health benefits, and therapeutic properties of avenanthramides: From skin protection to prevention and treatment of cerebrovascular diseases. Oxid. Med. Cell Longev..

[11] Cho S.S., Qi L., Fahey G.C., Klurfeld D. (2013). Consumption of cereal fiber, mixtures of whole grains and bran, and whole grains and risk reduction in type 2 diabetes, obesity, and cardiovascular disease. Am. J. Clin. Nutr..

[12] Aune D., Norat T., Romundstad P., Vatten L.J. (2013). Whole grain and refined grain consumption and the risk of type 2 diabetes: A systematic review and dose-response meta-analysis of cohort studies. Eur. J. Epidemiol..

[13] Fogelholm M., Anderssen S., Gunnarsdottir I., Lahti-Koski M. (2012). Dietary macronutrients and food consumption as determinants of long-term weight change in adult populations: A systematic literature review. Food Nutr. Res..

[14] Wang Q., Ellis P.R. (2014). Oat β-glucan: Physico-chemical characteristics in relation to its blood-glucose and cholesterol-lowering properties. Br. J. Nutr..

[15] Maki K.C., Beiseigel J.M., Jonnalagadda S.S., Gugger C.K., Reeves M.S., Farmer M.V., Kaden V.N., Rains T.M. (2010). Whole-grain ready-to-eat oat cereal, as part of a dietary program for weight loss, reduces low-density lipoprotein cholesterol in adults with overweight and obesity more than a dietary program including low-fiber control foods. J. Am. Diet Assoc..

[16] Beck E.J., Tosh S.M., Batterham M.J., Tapsell L.C., Huang X.F. (2009). Oat beta-glucan increases postprandial cholecystokinin levels, decreases insulin response and extends subjective satiety in overweight subjects. Mol. Nutr. Food Res..

[17] Rebello C.J., Chu Y.F., Johnson W.D., Martin C.K., Han H., Bordenave N., Shi Y., O’Shea M., Greenway F.L. (2014). The role of meal viscosity and oat (-glucan characteristics in human appetite control: A randomized crossover trial. Nutr. J..

[18] El Khoury D., Cuda C., Luhovyy B.L., Anderson G.H. (2012). Beta glucan: Health benefits in obesity and metabolic syndrome. J. Nutr. Metab..

[19] Food and Drug Administration (FDA), Code of Federal Regulations. https://www.accessdata.fda.gov/scripts/cdrh/cfdocs/cfcfr/CFRSearch.cfm?fr=101.81.

[20] Fulgoni V.L., Chu Y., O’Shea M., Slavin J.L., DiRienzo M.A. (2015). Oatmeal consumption is associated with better diet quality and lower body mass index in adults: The National Health and Nutrition Examination Survey (NHANES), 2001–2010. Nutr. Res..

[21] O’Neil C.E., Nicklas T.A., Fulgoni V.L., DiRienzo M.A. (2015). Cooked oatmeal consumption is associated with better diet quality, better nutrient intakes, and reduced risk for central adiposity and obesity in children 2–18 years: NHANES 2001–2010. Food Nutr. Res..

[22] National Health and Nutrition Examination Survey. https://www.cdc.gov/nchs/nhanes/index.htm.

[23] Rhodes D.G., Adler M.E., Clemens M.E., Moshfegh A.J. (2017). What we eat in America food categories and changes between survey cycles. J. Food Comp. Anal..

[24] Krebs-Smith S.M., Pannucci T.E., Subar A.F., Kirkpatrick S.I., Lerman J.L., Tooze J.A., Wilson M.M., Reedy J. (2018). Update of the Healthy Eating Index: HEI-2015. J. Acad. Nutr. Diet..

[25] Overview of Food Patterns Equivalent Database. https://www.ars.usda.gov/northeast-area/beltsville-md-bhnrc/beltsville-human-nutrition-research-center/food-surveys-research-group/docs/fped-overview/.

[26] US Department of Agriculture National Agriculture Library: National Nutrient Database for Standard Reference. http://ndb.nal.usda.gov/.

[27] U.S. Department of Agriculture, Agricultural Research Service 2014. USDA Food and Nutrient Database for Dietary Studies 2011–2012. http://www.ars.usda.gov/ba/bhnrc/fsrg.

[28] U.S. Department of Agriculture, Agricultural Research Service 2016. USDA Food and Nutrient Database for Dietary Studies 2013–2014. http://www.ars.usda.gov/nea/bhnrc/fsrg.

[29] Hiza H.A., Casavale K.O., Guenther P.M., Davis C.A. (2013). Diet quality of Americans differs by age, sex, race/ethnicity, income, and education level. J. Acad. Nutr. Diet.

[30] Reedy J., Krebs-Smith S.M., Bosire C. (2010). Evaluating the food environment: Application of the Healthy Eating Index-2005. Am. J. Prev. Med..

[31] Diet quality of older Americans in 1994–1996 and 2001–2002 as measured by the Healthy Eating Index-2005. https://www.cnpp.usda.gov/sites/default/files/nutrition_insights_uploads/Insight41.pdf.

[32] Fulgoni V.L., Keast D.R., Drewnowski A. (2009). Development and validation of the Nutrient-rich Foods Index: A tool to measure nutritional quality of foods. J. Nutr..

[33] Nicklas T.A., O’Neil C.E., Fulgoni V.L. (2012). Diet quality is inversely related to cardiovascular risk factors in adults. J. Nutr..

[34] Chiuve S., Fung T., Rimm E., Hu F., McCullough M., Wang M., Stampfer M.J., Willett W.C. (2012). Alternative dietary indices both strongly predict risk of chronic disease. J. Nutr..

[35] Reedy J., Mitrou P.N., Krebs-Smith S.M., Wirfält E., Flood A., Kipnis V., Leitzmann M., Mouw T., Hollenbeck A., Schatzkin A., Subar A.F. (2008). Index-based dietary patterns and risk of colorectal cancer: The NIH-AARP Diet and Health Study. Am. J. Epidemiol..

[36] O’Neil C.E., Nicklas T.A., Rampersaud G.C., Fulgoni V.L. (2011). One hundred percent orange juice consumption is associated with better diet quality, improved nutrient adequacy, and no increased risk for overweight/obesity in children. Nutr. Res..

[37] Harnack L., Walters S.A., Jacobs D.R. (2003). Dietary intake and food sources of whole grains among US children and adolescents: Data from the 1994–1996 Continuing Survey of Food Intakes by Individuals. J. Am. Diet Assoc..

[38] O’Neil C.E., Nicklas T.A., Zanovec M., Cho S.S., Kleinman R. (2011). Consumption of whole grains is associated with improved diet quality and nutrient intake in children and adolescents: The National Health and Nutrition Examination Survey 1999–2004. Public Health Nutr..

[39] McGill C.R., Fulgoni V.L., Devareddy L. (2015). Ten-year trends in fiber and whole grain intakes and food sources for the United States population: National Health and Nutrition Examination Survey 2001–2010. Nutrients.

[40] Albertson A.M., Reicks M., Joshi N., Gugger C.K. (2016). Whole grain consumption trends and associations with body weight measures in the United States: Results from the cross-sectional National Health and Nutrition Examination Survey 2001–2012. Nutr. J..

[41] Zanovec M., O’Neil C.E., Cho S.S., Kleinman R.E., Nicklas T.A. (2010). Relationship between whole grain and fiber consumption and body weight measures among 6- to 18-year-olds. J. Pediatr..

